# Evaluation of Glutathione-S-Transferase P1 Polymorphism and its Relation to Bone Mineral Density in Egyptian Children and Adolescents with Beta-Thalassemia Major

**DOI:** 10.4084/MJHID.2016.004

**Published:** 2016-01-01

**Authors:** Seham M. Ragab, Eman A. Badr, Ahmed S. Ibrahim

**Affiliations:** 1 Departments of Pediatrics, Faculty of Medicine, Menoufia University, NaserStreet, Shebeen El-koom, Menoufia, Egypt.; 2 Medical Biochemistry, Faculty of Medicine, Menoufia University, NaserStreet, Shebeen El-koom, Menoufia, Egypt.

## Abstract

**Background:**

Osteoporosis is a major complication of beta thalassemia major (TM). Increased oxidative stress and its controlling genes were linked to osteoporosis. Ile105 Val variant is a functional polymorphism of Glutathione S-transferase P1 (GSTP1), with reduced anti-oxidative property. No data are available about this variant or its association with osteoporosis among thalassemia patients yet.

**Objectives:**

To investigate Ile105Val polymorphism and its possible association with bone mineral density (BMD) values in a group of TM children.

**Methods:**

Thirty five TM children and 30 age and sex matched healthy controls were included. Liver and renal functions, serum ferritin, calcium, phosphorous, alkaline phosphatase and osteocalcin were assayed. BMD was determined by DXA with calculation of Z-scores at lumbar spine (LS) and femoral neck (FN). Height for age Z- score (HAZ) adjusted BMD Z-scores were calculated. GSTP1 Ile105Val polymorphism was studied by polymerase chain reaction-restriction fragment length polymorphism.

**Results:**

The relative frequency of 105 Val allele was significantly higher in TM patients than the controls (p<0.0001). Significant association between genotype subgroups and BMD parameters was detected. Compared to wild homozygotes, polymorphic homozygotes had lower LS-BMD (p =0.029), LS-BMD Z –score (p=0.008 ), LS- BMD _haz_ - Z-score (p=0.011), FN- BMD (p= 0.001), FN- BMD Z –score (p=0.02) and FN-BMD _haz_ - Z-score (p=0.001). They exhibited higher osteocalcin levels compared to heterozygotes and wild homozygotes (p=0.012, p=0.013, respectively).

**Conclusion:**

Ile105Val polymorphism was frequent among TM patients and could increase their susceptibility to reduced BMD. Large sample studies are required to confirm these findings.

## Introduction

Thalassemia major (TM) is the severest form of beta (β)-thalassemia. It is characterized by life-threatening anemia and iron overload.^1^ As a result of regular blood transfusions and increased compliance with iron chelation therapy, the life expectancy of β-thalassemia patients has greatly improved over the last years. However, this improvement is often accompanied by a series of grave complications including osteoporosis.^2^

Osteoporosis is a skeletal disease characterized by low bone mineral density (BMD) and deterioration of bone tissue micro-architecture with increased fracture risk.^3^

Thalassemia osteopathy is multi-factorial and culminates in a state of increased bone turnover with excessive bone resorption and remodeling. Hormonal deficiency, bone marrow expansion, high iron stores, deferoxamine toxicity, small body size, low baseline hemoglobin (Hb), delayed puberty and calcium/vitamin D deficiency are important risk factors.^4^

However, BMD is a complex quantitative trait that is genetically controlled in 50–90% of the cases according to twin and family studies.^5^

Genetic factors implication in osteoporosis among thalassemia patients has become a topic of widespread interest during the last decade. Polymorphisms of several genes, known to influence BMD, including collagen type I A1 (COLIA1),^6^ vitamin D receptor (VDR)^7^ and transforming growth factor-beta (TGF-β)^8^ have been previously investigated among thalassemia patients. However, the results were controversial.^9^

Glutathione S-transferases (GSTs) are a superfamily of genes whose gene products are enzymes responsible for catalyzing the bio-transformation of a variety of electrophilic compounds. Thus, they perform a pivotal role in the detoxification of activated metabolites of pro-carcinogens.^10^

GSTP1 -a pi class of the GST enzyme family- is the most prevalent isoform in non-hepatic tissues.^11^ Its gene is located on chromosome 11q13.^12^ Ile105Val polymorphism is a single nucleotide polymorphism in GSTP1 gene, caused by substitution of isoleucine for valine at amino acid codon 105. This substantially diminishes the enzyme activity and reduces its effective detoxification capacity.^13^

There is a growing body of evidence associating increased oxidative stress and low circulating antioxidants levels with reduced BMD and osteoporosis.^14^–^16^

Few studies have addressed the association between osteoporosis and polymorphisms of genes coding for enzymes involved in the anti-oxidative defense system.^17^–^20^

However, the published data about the association between GSTP1 polymorphism and osteoporosis are so scarce. There are no reports about this polymorphism among thalassemia patients. Therefore, this study aimed to investigate the frequency of GSTP1 (Ile105Val) polymorphism and its possible association with BMD values in a group of Egyptian children and adolescents with β-TM.

## Materials and Methods

### Study population

This cross sectional case-control study included 35 children and adolescents with TM (23 males and 12 females) who were recruited from the regular attendants of the Pediatric Hematology Clinic, Menoufia University Hospital, Egypt. Their ages ranged from 10–18 years with a mean age of 13.74 ±3.31 years. They were on regular packed red cell transfusion since infancy to maintain pre-transfusion hemoglobin (Hb) above 7.5 gm/dl and post transfusion Hb above 10gm/dl. Although, out of the international recommendation for TM transfusion, this regimen was the applicable one according to packed RBCs availability in our center and the compliance degree of our patients. Patients with diabetes mellitus (DM), abnormal thyroid functions, abnormal renal functions, serological evidence of hepatitis B or C and those under hormonal replacement therapy were excluded.

Thirty (15 males and 15 females), age, sex and ethnicity matched healthy children were involved as a control group. Their ages ranged from 10–18 years with a mean age of 14.33±2.66 years. They had normal complete blood count (CBC), Hb electrophoresis with no previous history of anemia, blood transfusion, liver or renal disease or family history of hemolytic anemia. They had been randomly selected from children presenting to our general outpatient clinic for follow up or with non-specific complaints.

The included adolescents ( 21 in the patient group and 22 in the controls) were non-smokers.

All included children were of native Egyptian ethnicity, who were born and lived in Menoufia governorate (one of Nile Delta governorates of North Egypt).

The study was conducted between July 2012 and December 2014. Informed consent was taken from the legal guardians of the included children before participation and ethical clearance from Faculty of Medicine, Menoufia University ethical committee was obtained.

Included patients were subjected to detailed history and thorough clinical examination. Clinico-demographic data were collected including history of chelation during the last year and vitamin D and calcium supplementation. Special emphasis was given for the presence of bone pain and the history bone fractures. All patients were under regular chelation therapy. Twenty two children (62.9%) were on regular subcutaneous deferoxamine (DFO) infusion (30–50 mg/kg/day, 5 days/week), 7(20 %) were on oral deferasirox (20–30 mg/kg/day) and 6 children (17.1%) were on oral deferiprone (75–100 mg/kg/day). Their compliance for chelation therapy was 60–70% ( mean of 64.4± 3.8 %, median of 65% ). According to our national recommended protocol for thalassemia management, all patients were supplemented with vitamin D (400 IU/day) and calcium (500 mg/day). History of splenectomy was documented in 10 patients (28.6%).

For each participant, body weight and height were measured by the standard methods with estimation of body mass index (BMI = weight in kg/height in m^2^). Height, weight and BMI for age Z-scores were calculated using the 2000 growth charts from the Centers for Disease Control and Prevention.^21^ Sexual maturity stage was assessed according to the criteria of Tanner.^22^

### Bone mineral density

BMD evaluation was performed using dual energy X-ray absorptiometry (DXA) (Norland–XR-46, USA, version 3.9.6/2.3.1) at lumbar spines (LS) (L1–L4) and femoral neck (FN). The BMD, results were converted to age- and gender-specific z scores based on the normative reference data for BMD in Egyptian children.

Due to the considerable height deficits for TM patients, BMD z -scores were then adjusted for height-for-age z score (HAZ) using the equations provided by Zemel et al.^23^ According to the Pediatric Position Development Conference (PDC) of The International Society of Clinical Densitometry (ISCD),^24^ BMD Z -scores of less than or equal to - 2, without a clinically significant fracture history, was defined as low BMD for chronologic age. While, osteoporosis was defined as BMD Z-scores of less than or equal to - 2 with a clinically significant fracture history with the term of osteopenia is no longer be used.

### Laboratory investigations

Laboratory investigations included CBC using AC920 Autocounter (pre-transfusion values were considered for the patient group) and quantitative colorimetric measurement of serum alanine aminotransferase (ALT), aspartate aminotransferase (AST), blood urea, serum creatinine, calcium (Ca), phosphorus (Ph) and total alkaline phosphatase (TALK). Serum ferritin was estimated by a two-site immune-luminometric assay (Byk- Sangtec Diagnostica). The mean serum ferritin level in the previous 2 years was calculated (on the average of four determinations per year) for each patient. Determination of osteocalcin in the serum was performed by the Enzyme-Linked Immunosorbant Assay (ELISA) method (Human Osteocalcin ELISA, Diagnostic Systems Laboratories, Inc., Webster, TX, USA).

### GSTP1 genotyping

Genomic DNA was extracted from peripheral blood leukocytes using the QIAamp DNA Blood Mini Kit (Qiagen Hilden, Germany). DNA eluted in buffer AE was stored at −20° C for polymerase chain reaction (PCR).

The GSTP1 Ile105Val substitution was detected by allele specific PCR restriction fragment length polymorphism (PCR-RFLP).^25^

**Figure 1:**
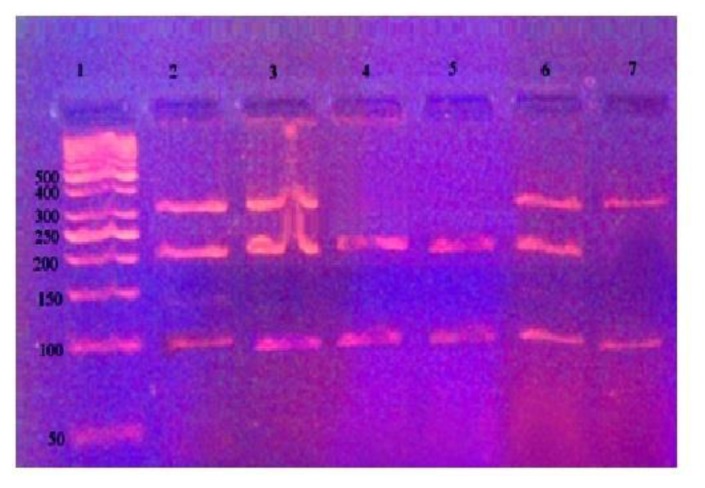
The agarose gel electrophoresis for Ile/Val polymorphism after digestion by Alw261;Lane 1 indicates DNA ladder (50 bp);Lanes 2, 3 and 6 indicate GSTP1 Ile/Val (AG) polymorphism (329bp, 216bp and 113 bp);Lanes 4,5 indicate GSTP1 Val/Val(GG) polymorphism (216bp and113 bp). Lane 7 indicates GSTP1 Ile/Ile (AA) polymorphism (329bp and 113 bp).

PCR was carried out to a total volume of 25μl of solution containing 10 × PCR buffer [16.6 mmol/l (NH4)2SO4, 20.0mmol/l MgCl2, pH 8.8, 1.2 μl Dimethyl sulfoxide (DMSO), 1.2 μl Dithiothreitol (DTT); Genecraft, Germany)], 100 ng of genomic DNA, 1 U of Taq DNA polymerase(Genecraft, Germany), 200 μmol/l deoxynucleoside triphosphates(dNTPS); (Stratagene, USA) and 25 pmol of *GSTP1* primers(forward primer; 5-GTA GTT TGC CCA AGGTCA AG-3 and reverse primer 5-AGC CAC CTG AGG GG TAAG -3., Midland, Texas). PCR amplification was performed in a programmable Perkin Elmer thermal cycler 2400 (USA) as follows: 94°C for 3 minutes followed by 5 cycles at 94°C for 15 seconds, 64°C for 30 seconds and 72°C for 1 minute during which the annealing temperature decreased by 1°C for each cycle. This was followed by 30 cycles of denaturation at 94°C for 15 seconds, annealing at 59°C for 30 seconds and extension at 72°C for 1 minute followed by a final polymerization step at 72 °C for5 minutes. A negative control (PCR without template) was included in each set of PCR reactions. The amplification products were separated by electrophoresis through 3% agarose gel stained with ethidium bromide with one band was observed (442bp). The PCR product of the GSTP1 gene was then digested by 5 U Alw261 restriction enzyme (Fermentas). The mixture was incubated for 4 hours at 37°C then 10μl of the products were loaded into 3% agarose gel containing ethidium bromide for electrophoresis and was visualized under ultraviolet trans-illuminator. The digestion products resulted in 329 and 113bp bands for Ile/Ile (AA; wild homozygote), 329, 216 and113bp bands for Ile/Val (AG; heterozygote for the polymorphism) and 216 and 113bp bands for Val/Val (GG; homozygote for the polymorphism) genotypes (Figure 1).

## Statistical Analysis

The data were processed on an IBM-PC compatible computer using SPSS version 16 (SPSS Inc., Chicago, IL, USA). Continuous variables were presented as mean ± SD, while for categorical variables, numbers (%) were used. Allele frequencies were estimated by the gene counting. Chi-square (χ2) test was used for comparison of the categorical variables. Student’s t- and ANOVA tests were used to compare continuous parametric variables in two and more than two groups, respectively. While Mann-Whitney (U) and Kruskal–Wallis tests were used for comparing non parametric variables in two and more than two groups, respectively. The least significant difference test (for parametric variables) and Tukey’s honest significant difference (HSD) test (for non-parametric variables) were applied for comparisons between individual groups when appropriate. Pearson and Spearman Rank correlation coefficients were applied for parametric and non-parametric data respectively. All tests were two-tailed and p value <0.05 was considered statistically significant.

## Results

### Patients characteristics

Weight Z –score <- 2 was found in 13 patients (37.1%), height Z-score < -2 was found in 11 patients (31.4%) while BMI Z –score <- 2 was found in 8 patients (22.9%). All patients had history of bone pain. Long bone fractures of the lower limb were documented in 2 patients (5.7%) and were related to minor trauma.

Children with TM had significantly lower body weight, height, BMI and their Z-scores compared to the controls. Delayed puberty was found in 57.14% of the patients (20/35). Patients had significantly lower pre-transfusion Hb, significantly higher ALT, ALK, the mean yearly ferritin and osteocalcin levels without significant difference in AST, blood urea, serum creatinine, calcium or phosphorous compared to the controls (Table 1). Hypocalcemia was diagnosed in 3 patients (8.57%) and hyperphosphatemia was found in 2 patients (5.71%). These 5 patients had hypo-parathyrodism.

DXA study revealed significant lower BMD values and Z –scores as well as BMD _haz_ Z-scores at both LS and FN in TM patients compared to the controls (p<0.0001) (Table 1). Low for chronological age BMD Z –scores at LS and FN were found in 88.57% (31/35) and 57.14% (20/35) of the patients respectively. Applying HAZ adjusted values, low for chronological age BMD_haz_ Z scores were found in 51.43% (18/35) and in 40% (14/35) of the patients at LS and FN respectively. Osteoporosis was diagnosed in 2 male patients (5.7%). There was no significant difference between males and females in any of the tested parameters including DXA scan parameters (p>0.05) (Table 3).

**Figure 2:**
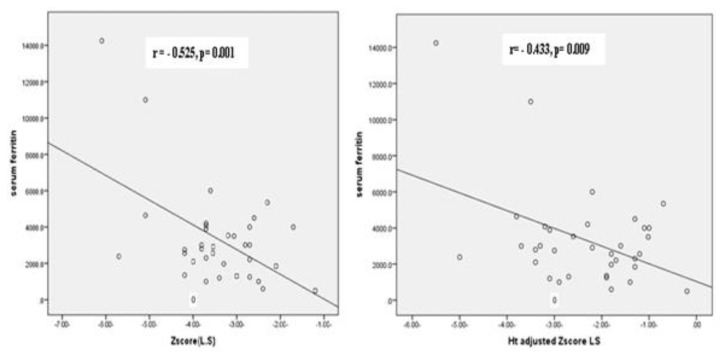
**A.** Correlation between BMD Z score LS and the mean yearly serum ferritin among the patient group. **B.** Correlation between BMD _HAZ_ -Z LS and the mean yearly serum ferritin among the patient group.

Both BMD Z-score at LS and its HAZ adjusted values had significant inverse correlation with serum ferritin (r = −0.525, p= 0.001 and r= −0.433, p= 0.009, respectively; Figure 2). Both FN-BMD Z-score and its HAZ adjusted values had a negative correlation trend with serum ferritin (r= −0.33, p=0.05).

### GSTP1 genotyping

The 105Val allele was found in 50.77% (33/65) of all studied children (the patients and the controls), with an allele frequency of 0.338 in the whole group, 0.51 in the patient group and 0.13 in the control group. The allele frequency and the polymorphism relative subgroups frequency (homozygotes and heterozygotes) were significantly prevalent in TM children compared to the controls (p<0.0001) (Table 1).

### Comparison of different GSTP1 genotypes

**Figure 3:**
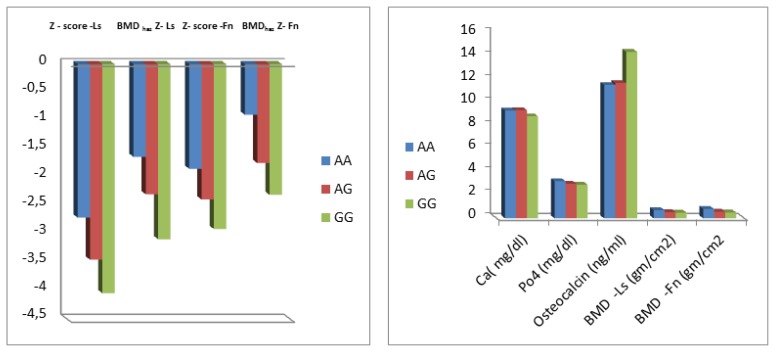
**A.** Comparison between different GSTP1 genotypes in DXA scan parameters among the patient group. **B.** Comparison between different GSTP1 genotypes in the studied laboratory parameters among the patient group.

Among children with TM, no significant difference was found in the clinical parameters between Ile105Val polymorphism genotype subgroups. Patients with Ile105Val polymorphism (homozygotes and heterozygotes) had significant lower BMD- LS, BMD LS-Z- score, FN- BMD and FN - BMD _haz_ Z-score (p<0.05) with modest significant lower BMD _haz_ Z-score at LS (p=0.05) than those without this polymorphism. No significant difference was found in any of the tested biochemical parameters between patients with Ile105Val polymorphism and those who do not carry this polymorphism. Significant association was found between Ile105Val polymorphism genotype subgroups and BMD (p<0.05). Post hoc analysis revealed that heterozygotes had significant lower FN-BMD and FN-BMD _haz_ Z-score (p=0.006 and 0.02, respectively) with modest lower LS- BMD (p=0.05) and LS- BMD Z-score (p=0.08) compared to the wild homozygotes. In comparison to wild homozygotes, Ile105Val polymorphism homozygotes had significant lower LS-BMD (p =0.029), LS-BMD Z –score (p=0.008 ), LS- BMD _haz_ Z-score (p=0.011), FN- BMD (p= 0.001), FN- BMD Z –score (p=0.02) and FN- BMD _haz_ Z-score (p=0.001). Polymorphism homozygotes also had a trend of lower LS- BMD _haz_ Z-score than the heterozygotes (p=0.09). Osteocalcin level was significantly higher in the polymorphic homozygotes compared to heterozygotes and wild homozygotes (p=0.012 and p=0.013, respectively). No significant difference was observed regarding the other tested parameters (Table 2, Figure 3).

## Discussion

Several lines of evidence have found a tight association between oxidative stress and its genetic control and reduced BMD.^14^–^20^

For the first time, we investigated the frequency of GSTP1 Ile105Val polymorphism in a group of children with TM compared to age, sex and race matched healthy non-related Egyptian children.

Distinct ethnic differences exist in the frequency of this polymorphism.^26^

The polymorphic allele was found in 50.77% (33/65) of all included children. The detected allele frequency of 0.338 and genotype distribution (49.23%, 33.85% and 16.92% for Ile/Ile, Ile/Val and Val/Val genotypes, respectively) are more or less similar to what was reported among Africans (0.343 for the allele frequency and the 45.14%, 41.09% and 13.77 for Ile/Ile, Ile/Val and Val/Val genotypes, respectively.^27^,^28^

Little data are available about Ile 105Val polymorphism among Egyptians. In 300 Egyptians subjects (112 with type 2 DM and 188 healthy), this polymorphism was found in 23.33 % of the studied group (70 out of 300); all were heterozygotes with an allele frequency of 0.116.^29^ The difference between these results and that of our study could be related to the difference in the sample size or the included primary disease category.

Ile 105Val polymorphism (both in hetero- and homozygous states) was significantly prevalent among TM patients than the controls. As far as we know, no similar studies were done among patients with thalassemia to compare. Nevertheless, this polymorphism was studied among patients with sickle cell anemia (SCA) with contradictory results. In a Brazilian study, significant higher frequency of 105Val polymorphism both in hetero- and homozygous states was found among SCA patients than the controls.^30^ However, similar allele frequencies and genotype distribution of this polymorphism were found in 50 Egyptian SCA patients and healthy controls in another study.^31^ Higher frequency of 105Val polymorphism among β-thalassemia patients detected in this work and among SCA patients in the previous Brazilian study^30^ compared to the healthy controls may suggest genetic linkage between, GSTP1 gene (11q13) and β-globin gene (11p15).

Oxidative stress related osteopathy may be related to several mechanisms. Reactive oxygen species (ROS) could antagonize Wnt signaling required for osteo-blastogenesis, thereby attenuating bone formation.^32^ Furthermore, hydrogen peroxide -induced oxidative stress inhibits osteoblastic differentiation via extracellular signal-regulated kinases (ERKs) and ERK dependent nuclear factor-κB (NF-κB) signaling pathway.^33^

GSTP1 iso-enzymes play a regulatory role in cellular signaling involved in controlling stress response.^34^ Its bone protective action is exerted through coordinated regulation of stress kinases (increase p38, ERK, and NF-κB activities together with suppression of JNK signaling ), thus contributing in protection against ROS -mediated bone cell apoptosis.^33^,^35^

Ile105Val polymorphism is located within the active site of the enzyme. This augments its importance in reducing enzyme activity with increased cell sensitization to free radical-mediated damage.^36^ Hence, it may be considered as a possible predisposing factor for ROS associated diseases like osteoporosis.

Thalassemia major is a disease of enhanced oxidative stress and high ROS levels, in which iron overload is the master player via the Fenton reaction.^37^

So, this disease is a good candidate for the influence of genetic polymorphism affecting the oxidative process especially Ile 105 Val polymorphism. To our knowledge, this study is the first attempt to evaluate the effect of this polymorphism with regard to BMD among thalassemia patients. It also has the novelty of application of HAZ adjusted BMD Z –scores as a more reliable measure for children with linear growth or maturation delay like TM children.^24^

Ile 105Val polymorphism was significantly prevalent among our studied TM children - with their high prevalence of low BMD- compared to the controls. In addition, the salient finding of this study was the presence of significant association between 105Val allele even in the heterozygous state and reduced BMD in these children. These findings could raise the assumption that this polymorphism plays a role in their predisposition to reduced BMD.

Osteocalcin is an important marker of bone turnover.^38^ Its detected high level among the studied TM patients could indicate that these patients had preserved bone formation despite severe bone destruction.^39^

Among the studied TM patients, 105Val allele in the homozygous state was significantly associated with high osteocalcin level. This finding could suggest a possible role of this polymorphism in enhancing bone turnover among thalassemia patients.

Data from the only similar published study performed on Slovenian women,^40^ revealed some consistent findings with this study. The authors reported significant association between Ile105Val genotype subgroups and osteocalcin levels in osteopenic post-menopausal women being significantly higher in 105Val polymorphism homozygotes compared to the wild homozygotes. In contrast to our results, the researchers detected non - significant increase in BMD values at all tested sites (LS and FN) in the heterozygotes with non-significant reduction of these values in the homozygotes of this polymorphism. Co-inheritance of 105Val and 114 Val alleles caused borderline significant lower BMD –FN values and increased osteocalcin concentrations compared to carriers lacking this combination.

The discordance between our results and that of the Slovenian study might be partly due to the difference in the included populations and to what is known that the 105 Val allele exhibits different activity, affinity and thermo-stability according to substrates.^41^ Furthermore, phenotypic expression of GSTP1 polymorphisms could be mediated by interactions with other polymorphic loci of other GST family genes.^40^

The underlying mechanisms of iron induced low BMD are not fully elucidated yet. In vitro data indicated that iron-induced bone damage was predominantly attributable to ROS mediated disequilibrium between bone formation and resorption.^42^

Although less reliable, serum ferritin is the wildly accepted parameter of iron overload among thalassemia patients in clinical practice.^43^

The results of this study revealed significant inverse correlation between LS-Z-score and its HAZ adjusted values with the mean yearly serum ferritin level indicating the contribution of iron overload on reduced BMD which comes in line with what was reported.^4^

Few previous studies have investigated the effect GSTM1 and GSTT1 genes polymorphisms on iron overload among thalassemia patients with controversial results^44^–^47^ without data for GSTP1 polymorphism in this regard.

No significant association was found between 105Val polymorphism and the mean yearly serum ferritin as an estimate for iron overload among the studied TM patients. So, we could assume that, the deleterious impact of 105Val allele on BMD among TM patients was not through favoring more iron overload, but it could be through enhancing iron generated ROS damaging effect.

Iron overload-induced hypogonadotropic hypogonadism with delayed puberty is another mechanism of osteoporosis in TM patients.^39^ In this work delayed puberty did not differ among different Ile105Val genotypes, a finding that excludes delayed puberty as a conflicting factor. Moreover, exclusion of smokers eliminates the confounding effect of smoking.

The small sample size and the single locality are the limitation of this study. So, our results could be considered preliminary.

Regarding the effect of ethnicity, it is known that, the vast majority of the Egyptians (98%) have the Egyptian (Arabs) ethnicity, while other ethnicities constitute the minority [Berber, Nubian, Bedouin, and Beja about 1%, Greek, Armenian, other European (primarily Italian and French) about 1%]. All participants of this study (the patients and the controls) were ethnically homogeneous as they are native Peasant Egyptians. Moreover, all of them originated from the same environment and have similar lifestyles. This could reduce the possibility of ethnic difference influence.

## Conclusions

GPT1 Ile 105Val polymorphism was significantly prevalent among the studied TM children compared to the controls. This polymorphism was associated with a remarkable reduction of BMD and elevation of osteocalcin levels especially among the homozygotes. This could highlight the significant relevance of this polymorphism as a candidate gene for osteoporosis in TM patients. The results affirm the hypothesis that ineffective ROS detoxification may be a risk for decreased BMD values. Multicenter studies with large samples and on other ethnic populations are required to confirm these findings.

## Figures and Tables

**Table 1:** Clinical characteristics, laboratory parameters and GSTP1 analysis of studied groups.

Variable	Patients (n=35)	Control (n=30)	P-value
Age (years)	13.7±3.3	14.3±2.6	0.43
Sex male, n (%)	23(65.7)	15(50)	0.22
Weight (Kg)	36.22±10.82	57.46±12.34	<0.0001
Weight for age Z- score(WAZ)	−1.5±0.67	0.78±0.36	<0.0001
Height (Cm)	142.06±14.6	156.6±11.28	0.001
Height for age Z- score(HAZ)	−1.25±0.87	−0.033±0.26	<0.0001
BMI	17.9±3.8	23.1±2.5	<0.0001
BMI for age Z- score	−1.25±0.87	−0.033±0.26	<0.0001
Puberty, n (%)			
Normal	15(42.9)	30(100)	<0.0001
Delayed	20(57.1)	0(0.0)	
Hb (gm/dl )	7.6±0.52	12.82±0.67	<0.0001
ALT (U/l)	41.17±29.82	16.33±0.64	<0.0001
AST (U/l)	42.57±32.53	27.54±3.67	0.727
Blood urea (mg/dl)	16.71±3.43	16.67±3.03	0.95
Serum creatinine (mg/dl)	0.52±0.12	0.55±0.15	0.33
Serum Ca ( mg/dl)	9.21±0.7	9.52±0.37	0.09
Serum Ph ( mg/dl)	3.02±0.45	2.82±0.49	0.1
TALP (U/l)	764.03±75.03	94.27±20.35	<0.0001
Serum Ferritin (ng/ml )	3329.6±2707.7	99.67±16.28	<0.0001
Osteocalcin ( ng/ml)	13.07±2.87	4.63±1.13	<0.0001
BMD -LS (gm/cm2)	0.57±0.20	1.53±0.47	<0.0001
Z - score -LS	−3.42±1.04	1.23±0.76	<0.0001
BMD _haz_ Z- LS	−2.34±1.19	1.35±0.7	<0.0001
BMD -FN (gm/cm2)	0.59±0.21	1.54±0.46	<0.0001
Z- score -FN	−2.39±0.96	1.24±0.72	<0.0001
BMD_haz_ Z- FN	−1.67±0.96	1.35±0.75	<0.0001
Allele frequency			
A	0.49	0.87	<0.0001
G	0.51	0.13	
Ile 105 Val allele, n (%)			
Polymorphic (G allele )	27(77.1)	6(20)	<0.0001
Non-polymorphic (A allele )	8(22.9)	24(80)	
Ile 105 Val genotype, n (%)			
Wild homozygotes (AA)	8 (22.9)	24 (80)	<0.0001
Heterozygotes (AG)	18 (51.4)	4 (13.3)	0.001
Homozygotes (GG)	9 (25.7)	2 (6.7)	0.04

BMI; body mass index; Hb; hemoglobin; ALT; alanine aminotransferase; AST; aspartate aminotransferase; Ca; calcium; Ph; phosphorous; TALK; total alkaline phosphatase; BMD; bone mineral density; LS; lumbar spine; FN; femoral neck.

**Table 2:** Comparison between different GSTP1 genotypes in patients with TM regarding to different parameters.

	Patients with the polymorphic allele (AG and GG ) (n=27)	AA (n=8)	AG (n=18)	GG (n=9)
Age (yr)	13.7±3.5	14.0±2.9	12.9±3.4	15.1±3.3
Male, n (%)	19 (70.4)	4(50)	12(66.7)	7(77.8)
Weight (kg)	36.0±10.9	37.0±11.4	33.6±9.9	40.9±11.5
Weight for age Z-score(WAZ)	−1.47±0.7	−1.62±0.6	−1.34±0.75	−1.72±0.55
Height (cm)	142.48±14.42	140.62±16.13	139.5±12.954	148.44±16.12
Height for age Z-score (HAZ)	−1.26±0.79	1.21±1.15	−1.32±0.73	−1.16±0.96
BMI (kg/m2)	17.98±3.95	17.6±3.6	18.3±4.1	17.4±3.7
BMI for age Z-score	−0.57±1.13	−0.69±1.1	−0.31±1.13	−1.08±1
Puberty, n(%)				
Normal	11(40.7)	4(50)	8(44.4)	3(33.3)
Delayed	16(59.3)	4(50)	10(55.6)	6(66.7)
Hb(g/dl)	7.59±51	7.62±0.59	7.71±0.4	7.37±0.65
ALT (U/l)	39.25±27.23	47.62±38.79	38.39±30.03	41.0±22.1
AST (U/l)	39.7±27.25	52.25±47.35	39.78±29.57	39.56±23.55
Blood urea (mg/dl)	16.59±3.24	17.13±4.22	16.55±3.63	16.67±2.45
Serum creatinine (mg/dl)	0.52±0.13	0.53±0.12	0.53±0.11	0.49±0.17
Serum ferritin (ng/ml)	3581.9±2961.28	2478.1±1402.2	2958.5±1321.1	4828.7±4708.28
Ca( mg/dl)	9.17±0.68	9.32±0.8	9.35±0.56	8.82±0.79
Ph (mg/dl)	2.96±0.39	3.21±0.58	2.99±0.37	2.91±0.45
TALP( u/l)	770.4±75.8	782.8±65.8	765.7±85.3	744.1±62.3
Osteocalcin (ng/ml)	13.52±2.73	11.53±2.9	11.7±2.1	14.4±2.58&$
BMD -LS (gm/cm2)	0.530 ± 0.18	0.703 ±0.208*	0.541±0.177	0.493±0.197&
Z - score -LS	−3.64±1.02	−2.7±0.8*	−3.44±0.82	−4.03±1.3&
BMD _haz_ Z- LS	−2.55±1.2	−1.63± 0.88	−2.29 ± 1.12	−3.08± 1.26&
BMD -FN (gm/cm2)	0.560 ± 0.13	0.810±0.23**	0.580±0.14&	0.51±0.07&
Z- score -FN	−2.56±0.93	−1.84±0.85	−2.38±0.85	−2.9±1.04&
BMD_haz_ Z- FN	−1.93±0.85	−0.89 ±0.84*	−1.74 ± 0.76&	−2.3± 0.93&

BMI; body mass index; Hb; hemoglobin; ALT; alanine aminotransferase; AST; aspartate aminotransferase; Ca; calcium; Ph; phosphorous; TALK; total alkaline phosphatase; BMD; bone mineral density; LS; lumbar spine; FN; femoral neck.

* P<. 0.05 comparing AA versus (AG and G),

** P<. 0.0001 comparing AA versus (AG and GG),

& Significant compared with the wild homozygotes (AA),

$ Significant compared with heterozygotes (AG)

**Table 3:** Comparison between males and females thalassemic patients regarding to different parameters.

Variable	Males (n=23)	Females (n=12)	P-value
Age (years)	13.6±3.1	14.0±3.8	0.79
Weight (Kg)	37.3±10.9	34.2±10.9	0.41
Weight for age Z- score (WAZ)	−1.46±0.64	−1.43±0.71	0.92
Height (Cm)	143.9±14.8-	138.6±14.2	0.32
Height for age Z- score (HAZ)	1.3±0.78	−1.13±1.18	0.59
BMI	18.26±4.16	17.18±3.16	0.43
BMI for age Z- score	−0.47±1.2	−0.84±0.9	0.35
Puberty, n (%)			
Normal	11(52.2)	4(33.3)	0.41
Delayed	12(47.8)	8(66.7)	
Hb (gm/dl )	7.54±0.57	7.72±0.41	0.36
Serum Ca ( mg/dl)	9.2±0.63	9.21±0.85	0.97
Serum Ph ( mg/dl)	3.00±0.44	3.1±0.49	0.68
TALP (U/l)	762.4±82.3	767.2±62.1	0.86
Serum Ferritin (ng/ml )	3732.4±3180.5	2557.6±1200.3	0.33
Osteocalcin ( ng/ml)	13.3±2.6	12.6±3.4	0.53
BMD -LS (gm/cm2)	0.57±0.22	0.56±0.16	0.75
Z - score -LS	−3.5±1.2	−3.4±0.63	0.88
BMD _haz_ Z- LS	−2.4±1.4	−3.4±0.84	0.72
BMD -FN (gm/cm2)	0.60±0.23	0.59±0.19	0.42
Z- score -FN	−2.3±0.96	−2.6±0.97	0.41
BMD_haz_ Z- FN	−1.7±0.97	−1.6±0.93	0.89
BMD -LS			
Osteoporosis	2(8.7)	0(0)	
Low BMD	21(91.3)	10(83.5)	0.08
Normal	0(0)	2(5.7)	
BMD- FN			
Osteoporosis	2(8.7)	0(00	
Low BMD	13(56.5)	7(58.3)	0.56
Normal	8(34.8)	5(41.7)	
BMD _haz_ Z- LS			
Osteoporosis	2(8.7)	0(0)	
Low BMD	14(60.9)	4(34.3)	0.09
Normal	7(30.4)	8(66.7)	
BMD_haz_ Z- FN			
Osteoporosis	2(8.7)	0(0)	
Low BMD	9(39.1)	5(45.7)	0.57
Normal	12(52.2)	7(58.3)	

BMI; body mass index; Hb; hemoglobin; Ca; calcium; Ph; phosphorous; TALK; total alkaline phosphatase; BMD; bone mineral density; LS; lumbar spine; FN; femoral neck.
